# 4-[(4-Acetyl­phen­yl)amino]-2-methyl­idene-4-oxo­butanoic acid

**DOI:** 10.1107/S1600536814012562

**Published:** 2014-06-07

**Authors:** B. Narayana, Prakash S. Nayak, Balladka K. Sarojini, Jerry P. Jasinski

**Affiliations:** a Department of Studies in Chemistry, Mangalore University, Mangaloagangotri 574 199, India; b Department of Studies in Chemistry, Mangalore University, Mangalagangotri 574 199, India; c Department of Studies in Chemistry, Industrial Chemistry Section, Mangalore University, Mangalagangotri 574 199, India; d Department of Chemistry, Keene State College, 229 Main Street, Keene, NH 03435-2001, USA

## Abstract

In the title compound, C_13_H_13_NO_4_, the N—C(=O) bond length of 1.354 (2) Å is indicative of amide-type resonance. The dihedral angle between the mean planes of the benzene ring and oxo­amine group is 36.4 (3)°, while the mean plane of the 2-methyl­idene group is inclined by 84.2 (01)° from that of the oxo­amine group. In the crystal, classical O—H⋯O hydrogen bonds formed by the carb­oxy­lic acid groups and weak N—H⋯O weak inter­actions formed by the amide groups and supported by weak C—H⋯O inter­actions between the 2-methyl­idene, phenyl and acetyl groups with the carb­oxy­lic acid, oxo­amine and acetyl O atoms, together link the mol­ecules into dimeric chains along [010]. The O—H⋯O hydrogen bonds form *R*_2_^2^(8) graph-set motifs.

## Related literature   

For the pharmacological activity of amide derivatives, see: Galanakis *et al.* (2004 ▶); Kumar & Knaus (1993 ▶); Ban *et al.* (1998 ▶); Ukrainets *et al.* (2006 ▶), Lesyk & Zimenkovsky (2004 ▶); Gududuru *et al.* (2004 ▶). For related structures, see: Nayak *et al.* (2013*a* ▶,*b* ▶). For standard bond lengths, see: Allen *et al.* (1987 ▶).
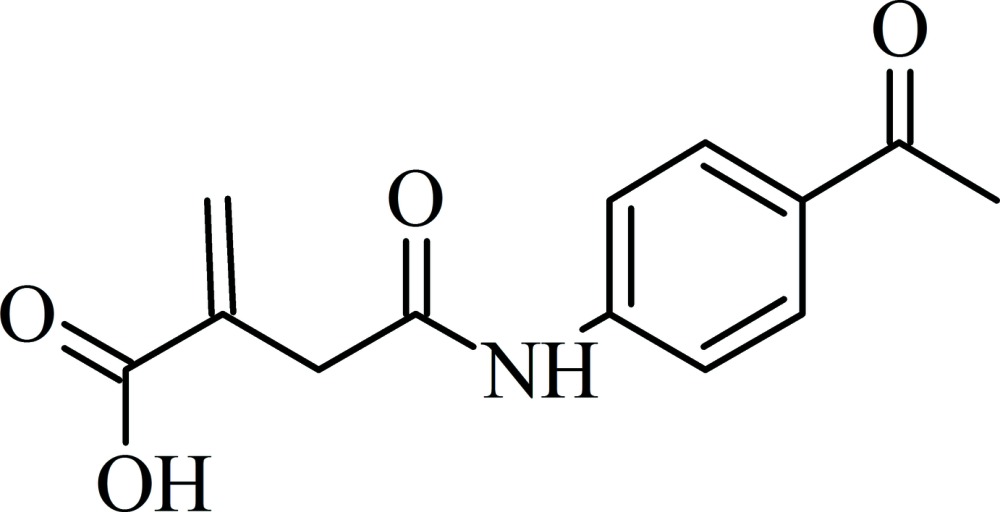


## Experimental   

### 

#### Crystal data  

C_13_H_13_NO_4_
*M* *_r_* = 247.24
Triclinic, 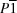

*a* = 5.0164 (5) Å
*b* = 5.2908 (4) Å
*c* = 21.8464 (18) Å
α = 92.833 (6)°
β = 90.315 (7)°
γ = 96.222 (7)°
*V* = 575.67 (8) Å^3^
*Z* = 2
Cu *K*α radiation
μ = 0.89 mm^−1^
*T* = 173 K
0.42 × 0.22 × 0.12 mm

#### Data collection  

Agilent Eos Gemini diffractometer
Absorption correction: multi-scan (*CrysAlis PRO* and *CrysAlis RED*; Agilent, 2012 ▶) *T*_min_ = 0.756, *T*_max_ = 1.000
3374 measured reflections
2168 independent reflections
1934 reflections with *I* > 2σ(*I*)
*R*_int_ = 0.025

#### Refinement  

*R*[*F* ^2^ > 2σ(*F* ^2^)] = 0.048
*wR*(*F* ^2^) = 0.134
*S* = 1.05
2168 reflections
176 parameters
H atoms treated by a mixture of independent and constrained refinement
Δρ_max_ = 0.31 e Å^−3^
Δρ_min_ = −0.29 e Å^−3^

### 

Data collection: *CrysAlis PRO* (Agilent, 2012 ▶); cell refinement: *CrysAlis PRO*; data reduction: *CrysAlis RED* (Agilent, 2012 ▶); program(s) used to solve structure: *SUPERFLIP* (Palatinus *et al.*, 2012 ▶); program(s) used to refine structure: *SHELXL2012* (Sheldrick, 2008 ▶); molecular graphics: *OLEX2* (Dolomanov *et al.*, 2009 ▶); software used to prepare material for publication: *OLEX2*.

## Supplementary Material

Crystal structure: contains datablock(s) I. DOI: 10.1107/S1600536814012562/zl2589sup1.cif

Structure factors: contains datablock(s) I. DOI: 10.1107/S1600536814012562/zl2589Isup2.hkl

Supporting information file. DOI: 10.1107/S1600536814012562/zl2589Isup3.cml

CCDC reference: 1005968

Additional supporting information:  crystallographic information; 3D view; checkCIF report

## Figures and Tables

**Table 1:** Hydrogen-bond geometry (Å, °)

*D*—H⋯*A*	*D*—H	H⋯*A*	*D*⋯*A*	*D*—H⋯*A*
O3—H3⋯O2^i^	0.97 (5)	1.66 (5)	2.6262 (17)	174 (4)
N1—H1⋯O1^ii^	0.88	2.29	3.1039 (17)	154
C5—H5*B*⋯O2^iii^	1.00 (3)	2.48 (3)	3.434 (2)	160 (2)
C7—H7⋯O1^ii^	0.95	2.56	3.254 (2)	130
C13—H13*A*⋯O4^iv^	0.98	2.50	3.465 (2)	167

Symmetry codes: (i) 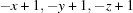; (ii) 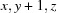; (iii) 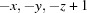; (iv) 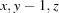.

**Fig. 1.**
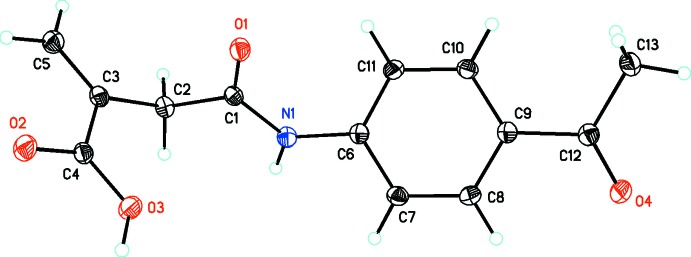
ORTEP drawing of C13H13NO4, showing the labeling scheme with 30% probability displacement ellipsoids.

**Fig. 2.**
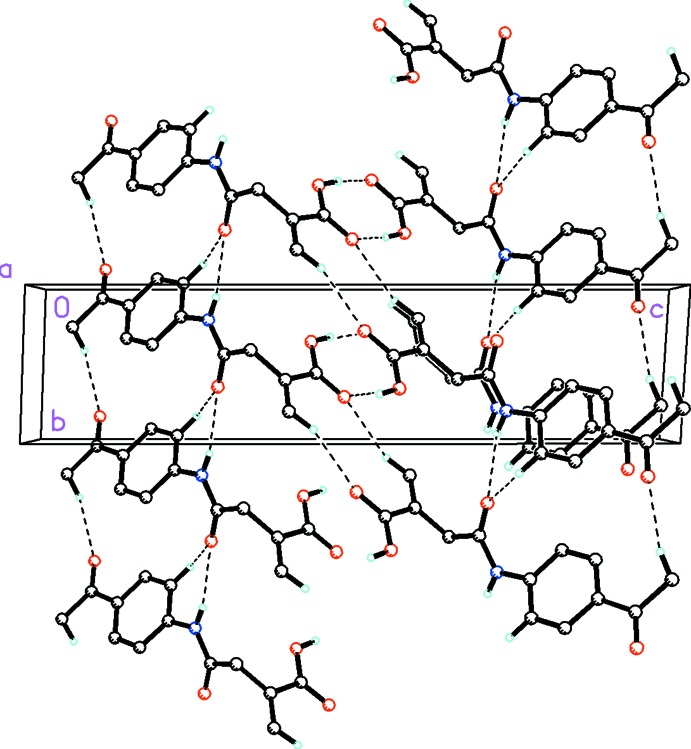
Molecular packing for C13H13NO4, viewed along the a axis. Dashed lines indicate O—H···O hydrogen bonds in an R22[8] motif format and weak N—H···O, C—H···O intermolecular interactions together linking the molecules into dimeric chains along [0 1 0]. H atoms not involved in hydrogen bonding have been removed for clarity.

**Fig. 3.**
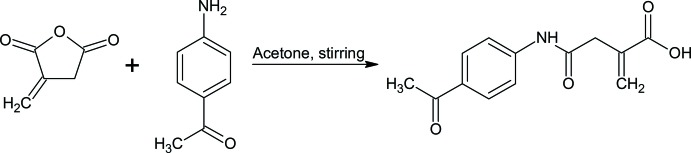
Synthesis of C13H13NO4.

## supplementary crystallographic information

### S1. Comment

Amide bonds play a major role in the elaboration and composition of biological systems, which are the main chemical bonds that link amino acid building blocks together to give proteins. Amide bonds are not limited to biological systems and are indeed present in a huge array of molecules, including major marketed drugs. Amide derivatives possessing anti-inflammatory (Galanakis *et al.*, 2004; Kumar *et al.*, 1993; Ban *et al.*, 1998), antimicrobial (Ukrainets *et al.*, 2006), anti-tubercular (Lesyk *et al.*, 2004) and antiproliferative (Gududuru *et al.*, 2004) activities are reported in the literature. Crystal structures of some amide derivatives related to the title compound include, viz., 4-(4-iodoanilino)-2-methylene-4-oxobutanoic acid and 4-(3-fluoro-4-methylanilino)-2-methylidene-4-oxobutanoic acid (Nayak *et al.*, 2013*a*,*b*). Hence in view of its potential pharmacological importance, the title compound 4-[(4-acetylphenyl)amino]-2-methylidene-4-oxobutanoic acid, C_13_H_13_NO_4_, was synthesized from 3-methylidenedihydrofuran-2,5-dione with good yields and its crystal structure is reported here.

In the title compound, The C=C bond is present as its *anti*-Saytzeff tautomer. The N–C(=O) bond length of 1.354 (2)A (A) is indicative of amide-type resonance (Fig. 1). All other bond lengths are in normal ranges (Allen *et al.*, 1987). In the crystal, classical O—H···O hydrogen bonds formed by the carboxylic groups and N—H···O weak intermolecular interactions formed by the amide groups and supported additionally by weak C—H···O intermolecular interactions between the 2-methylidene, phenyl and acetyl groups with the carboxylic, oxoamine and acetyl oxygen atoms (Table 1), together link the molecules into dimeric chains along [0 1 0] (Fig. 2). The O—H···O hydrogen bonds form R_2_^2^(8) graph-set motifs. The dihedral angle between the mean planes of the phenyl ring (C6–C10) and oxoamine group (C1/C2/O1/N1) is 36.4 (3)°, while the mean plane of the 2-methylidene group (C2–C5) is further inclined by 84.2 (1)° from that of the oxoamine group.

### S2. Experimental

3-Methylidenedihydrofuran-2,5-dione (0.112 g, 1 mmol) was dissolved in a 30 ml acetone and stirred at ambient temperature. 4-Aminoacetophenone (0.135 g, 1 mmol) in 20 mL acetone was added over 30 mins (Fig. 3). After sirring for 1.5 h the slurry was filtered. The solid was washed with acetone and dried to give the title compound, C_13_H_13_NO_4_. Single crystals were grown from methanol and toluene (1:1) mixture by the slow evaporation method (yield. 0.248 g, 87.32%, m.p.: 461–463 K).

### S3. Refinement

The OH atom was located by a difference map and refined isotropocally. All of the remaining H atoms were placed in their calculated positions and then refined using the riding model with Atom—H lengths of 0.95Å (CH), 0.98 - 1.00Å (CH_2_), 0.98Å (CH_3_) or 0.88Å (NH). Isotropic displacement parameters for these atoms were set to 1.2 (CH, CH_2_, NH) or 1.5 (CH_3_) times *U*_eq_ of the parent atom. Idealised Me was refined as a rotating group.

### Crystal data

**Table:**

C_13_H_13_NO_4_	*Z* = 2
*M**_r_* = 247.24	*F*(000) = 260
Triclinic, *P*1	*D*_x_ = 1.426 Mg m^−^^3^
*a* = 5.0164 (5) Å	Cu *K*α radiation, λ = 1.54184 Å
*b* = 5.2908 (4) Å	Cell parameters from 1583 reflections
*c* = 21.8464 (18) Å	θ = 6.1–71.3°
α = 92.833 (6)°	µ = 0.89 mm^−^^1^
β = 90.315 (7)°	*T* = 173 K
γ = 96.222 (7)°	Prism, colourless
*V* = 575.67 (8) Å^3^	0.42 × 0.22 × 0.12 mm

### Data collection

**Table:**

Agilent Eos Gemini diffractometer	2168 independent reflections
Radiation source: Enhance (Cu) X-ray Source	1934 reflections with *I* > 2σ(*I*)
Detector resolution: 16.0416 pixels mm^-1^	*R*_int_ = 0.025
ω scans	θ_max_ = 71.3°, θ_min_ = 4.1°
Absorption correction: multi-scan (*CrysAlis PRO* and *CrysAlis RED*; Agilent, 2012)	*h* = −5→6
*T*_min_ = 0.756, *T*_max_ = 1.000	*k* = −4→6
3374 measured reflections	*l* = −26→26

### Refinement

**Table:**

Refinement on *F*^2^	Primary atom site location: structure-invariant direct methods
Least-squares matrix: full	Hydrogen site location: mixed
*R*[*F*^2^ > 2σ(*F*^2^)] = 0.048	H atoms treated by a mixture of independent and constrained refinement
*wR*(*F*^2^) = 0.134	*w* = 1/[σ^2^(*F*_o_^2^) + (0.0796*P*)^2^ + 0.1341*P*] where *P* = (*F*_o_^2^ + 2*F*_c_^2^)/3
*S* = 1.05	(Δ/σ)_max_ < 0.001
2168 reflections	Δρ_max_ = 0.31 e Å^−^^3^
176 parameters	Δρ_min_ = −0.29 e Å^−^^3^
0 restraints	

### Special details

**Table:**

Geometry. All esds (except the esd in the dihedral angle between two l.s. planes) are estimated using the full covariance matrix. The cell esds are taken into account individually in the estimation of esds in distances, angles and torsion angles; correlations between esds in cell parameters are only used when they are defined by crystal symmetry. An approximate (isotropic) treatment of cell esds is used for estimating esds involving l.s. planes.

### Fractional atomic coordinates and isotropic or equivalent isotropic displacement parameters (Å^2^)

**Table:**

	*x*	*y*	*z*	*U*_iso_*/*U*_eq_	
O1	0.3166 (2)	0.3573 (2)	0.70834 (5)	0.0287 (3)	
O2	0.2328 (3)	0.2934 (2)	0.51283 (6)	0.0333 (3)	
O3	0.3843 (3)	0.6459 (2)	0.56835 (6)	0.0322 (3)	
H3	0.529 (9)	0.681 (8)	0.540 (2)	0.117 (15)*	
O4	1.3191 (3)	1.1439 (2)	0.91390 (6)	0.0347 (3)	
N1	0.3525 (3)	0.7850 (2)	0.72813 (6)	0.0238 (3)	
H1	0.2927	0.9262	0.7169	0.029*	
C1	0.2405 (3)	0.5648 (3)	0.70006 (7)	0.0212 (3)	
C2	0.0055 (3)	0.5917 (3)	0.65734 (7)	0.0236 (3)	
H2A	−0.1643	0.5631	0.6801	0.028*	
H2B	0.0205	0.7669	0.6428	0.028*	
C3	−0.0012 (3)	0.4045 (3)	0.60311 (7)	0.0230 (3)	
C4	0.2171 (3)	0.4458 (3)	0.55784 (7)	0.0235 (3)	
C5	−0.1886 (4)	0.2087 (3)	0.59389 (8)	0.0302 (4)	
H5A	−0.335 (4)	0.171 (4)	0.6228 (10)	0.030 (5)*	
H5B	−0.180 (5)	0.095 (5)	0.5563 (12)	0.051 (7)*	
C6	0.5569 (3)	0.8108 (3)	0.77393 (7)	0.0216 (3)	
C7	0.7359 (3)	1.0308 (3)	0.77582 (8)	0.0257 (4)	
H7	0.7233	1.1541	0.7461	0.031*	
C8	0.9319 (3)	1.0702 (3)	0.82088 (8)	0.0254 (4)	
H8	1.0534	1.2211	0.8218	0.030*	
C9	0.9549 (3)	0.8921 (3)	0.86518 (7)	0.0223 (3)	
C10	0.7744 (3)	0.6724 (3)	0.86265 (7)	0.0255 (4)	
H10	0.7873	0.5488	0.8923	0.031*	
C11	0.5764 (3)	0.6305 (3)	0.81778 (8)	0.0255 (4)	
H11	0.4545	0.4799	0.8168	0.031*	
C12	1.1696 (3)	0.9468 (3)	0.91332 (7)	0.0253 (4)	
C13	1.1922 (4)	0.7541 (3)	0.96093 (8)	0.0343 (4)	
H13A	1.2316	0.5927	0.9409	0.051*	
H13B	1.0227	0.7275	0.9830	0.051*	
H13C	1.3371	0.8164	0.9899	0.051*	

### Atomic displacement parameters (Å^2^)

**Table:**

	*U*^11^	*U*^22^	*U*^33^	*U*^12^	*U*^13^	*U*^23^
O1	0.0355 (7)	0.0221 (6)	0.0288 (6)	0.0058 (5)	−0.0079 (5)	−0.0017 (4)
O2	0.0372 (7)	0.0338 (7)	0.0269 (6)	−0.0017 (5)	0.0063 (5)	−0.0077 (5)
O3	0.0316 (7)	0.0325 (7)	0.0301 (7)	−0.0053 (5)	0.0045 (5)	−0.0039 (5)
O4	0.0393 (7)	0.0265 (6)	0.0362 (7)	−0.0048 (5)	−0.0101 (5)	0.0006 (5)
N1	0.0264 (7)	0.0206 (6)	0.0253 (7)	0.0068 (5)	−0.0025 (5)	−0.0003 (5)
C1	0.0228 (8)	0.0233 (8)	0.0178 (7)	0.0037 (6)	0.0017 (6)	0.0009 (6)
C2	0.0227 (8)	0.0266 (8)	0.0223 (8)	0.0067 (6)	−0.0002 (6)	−0.0004 (6)
C3	0.0238 (8)	0.0249 (8)	0.0212 (8)	0.0057 (6)	−0.0024 (6)	0.0023 (6)
C4	0.0258 (8)	0.0243 (7)	0.0206 (7)	0.0031 (6)	−0.0027 (6)	0.0009 (6)
C5	0.0304 (9)	0.0330 (9)	0.0262 (8)	−0.0001 (7)	0.0015 (7)	−0.0001 (7)
C6	0.0230 (8)	0.0213 (7)	0.0206 (7)	0.0047 (6)	0.0018 (6)	−0.0025 (6)
C7	0.0302 (9)	0.0217 (8)	0.0257 (8)	0.0038 (6)	0.0007 (6)	0.0038 (6)
C8	0.0264 (8)	0.0210 (7)	0.0281 (8)	−0.0001 (6)	0.0007 (6)	0.0016 (6)
C9	0.0240 (8)	0.0219 (7)	0.0212 (8)	0.0044 (6)	0.0016 (6)	−0.0026 (6)
C10	0.0328 (9)	0.0216 (7)	0.0218 (8)	0.0016 (6)	0.0001 (6)	0.0019 (6)
C11	0.0286 (8)	0.0218 (7)	0.0251 (8)	−0.0014 (6)	0.0000 (6)	0.0003 (6)
C12	0.0288 (8)	0.0224 (8)	0.0245 (8)	0.0038 (6)	−0.0006 (6)	−0.0030 (6)
C13	0.0427 (10)	0.0312 (9)	0.0281 (9)	0.0000 (7)	−0.0097 (7)	0.0027 (7)

### Geometric parameters (Å, º)

**Table:**

O1—C1	1.222 (2)	C6—C7	1.389 (2)
O2—C4	1.249 (2)	C6—C11	1.395 (2)
O3—H3	0.97 (5)	C7—H7	0.9500
O3—C4	1.288 (2)	C7—C8	1.380 (2)
O4—C12	1.216 (2)	C8—H8	0.9500
N1—H1	0.8800	C8—C9	1.397 (2)
N1—C1	1.354 (2)	C9—C10	1.392 (2)
N1—C6	1.420 (2)	C9—C12	1.497 (2)
C1—C2	1.523 (2)	C10—H10	0.9500
C2—H2A	0.9900	C10—C11	1.385 (2)
C2—H2B	0.9900	C11—H11	0.9500
C2—C3	1.504 (2)	C12—C13	1.504 (2)
C3—C4	1.485 (2)	C13—H13A	0.9800
C3—C5	1.328 (2)	C13—H13B	0.9800
C5—H5A	0.98 (2)	C13—H13C	0.9800
C5—H5B	1.00 (3)		
			
C4—O3—H3	118 (2)	C6—C7—H7	120.0
C1—N1—H1	116.8	C8—C7—C6	120.04 (15)
C1—N1—C6	126.47 (13)	C8—C7—H7	120.0
C6—N1—H1	116.8	C7—C8—H8	119.4
O1—C1—N1	123.53 (14)	C7—C8—C9	121.16 (15)
O1—C1—C2	121.41 (14)	C9—C8—H8	119.4
N1—C1—C2	115.05 (13)	C8—C9—C12	118.71 (15)
C1—C2—H2A	109.4	C10—C9—C8	118.15 (15)
C1—C2—H2B	109.4	C10—C9—C12	123.14 (14)
H2A—C2—H2B	108.0	C9—C10—H10	119.3
C3—C2—C1	111.32 (12)	C11—C10—C9	121.32 (15)
C3—C2—H2A	109.4	C11—C10—H10	119.3
C3—C2—H2B	109.4	C6—C11—H11	120.2
C4—C3—C2	116.83 (14)	C10—C11—C6	119.64 (15)
C5—C3—C2	123.91 (15)	C10—C11—H11	120.2
C5—C3—C4	119.26 (15)	O4—C12—C9	120.33 (15)
O2—C4—O3	123.36 (16)	O4—C12—C13	121.36 (16)
O2—C4—C3	120.94 (15)	C9—C12—C13	118.30 (14)
O3—C4—C3	115.70 (14)	C12—C13—H13A	109.5
C3—C5—H5A	122.5 (13)	C12—C13—H13B	109.5
C3—C5—H5B	118.9 (15)	C12—C13—H13C	109.5
H5A—C5—H5B	118.5 (19)	H13A—C13—H13B	109.5
C7—C6—N1	117.63 (14)	H13A—C13—H13C	109.5
C7—C6—C11	119.68 (15)	H13B—C13—H13C	109.5
C11—C6—N1	122.63 (14)		
			
O1—C1—C2—C3	−35.3 (2)	C6—N1—C1—C2	174.03 (14)
N1—C1—C2—C3	145.67 (14)	C6—C7—C8—C9	0.0 (3)
N1—C6—C7—C8	177.47 (14)	C7—C6—C11—C10	−0.1 (2)
N1—C6—C11—C10	−177.45 (14)	C7—C8—C9—C10	0.1 (2)
C1—N1—C6—C7	148.44 (16)	C7—C8—C9—C12	−179.32 (14)
C1—N1—C6—C11	−34.2 (2)	C8—C9—C10—C11	−0.2 (2)
C1—C2—C3—C4	−68.77 (17)	C8—C9—C12—O4	0.6 (2)
C1—C2—C3—C5	111.34 (18)	C8—C9—C12—C13	179.84 (15)
C2—C3—C4—O2	177.03 (14)	C9—C10—C11—C6	0.2 (3)
C2—C3—C4—O3	−3.0 (2)	C10—C9—C12—O4	−178.79 (16)
C5—C3—C4—O2	−3.1 (2)	C10—C9—C12—C13	0.4 (2)
C5—C3—C4—O3	176.90 (15)	C11—C6—C7—C8	0.0 (2)
C6—N1—C1—O1	−5.0 (3)	C12—C9—C10—C11	179.19 (14)

### Hydrogen-bond geometry (Å, º)

**Table:**

*D*—H···*A*	*D*—H	H···*A*	*D*···*A*	*D*—H···*A*
O3—H3···O2^i^	0.97 (5)	1.66 (5)	2.6262 (17)	174 (4)
N1—H1···O1^ii^	0.88	2.29	3.1039 (17)	154
C5—H5*B*···O2^iii^	1.00 (3)	2.48 (3)	3.434 (2)	160 (2)
C7—H7···O1^ii^	0.95	2.56	3.254 (2)	130
C13—H13*A*···O4^iv^	0.98	2.50	3.465 (2)	167

Symmetry codes: (i) −*x*+1, −*y*+1, −*z*+1; (ii) *x*, *y*+1, *z*; (iii) −*x*, −*y*, −*z*+1; (iv) *x*, *y*−1, *z*.
